# The ABCC6 Transporter as a Paradigm for Networking from an Orphan Disease to Complex Disorders

**DOI:** 10.1155/2015/648569

**Published:** 2015-08-18

**Authors:** Eva Y. G. De Vilder, Mohammad Jakir Hosen, Olivier M. Vanakker

**Affiliations:** ^1^Center for Medical Genetics, Ghent University Hospital, 9000 Ghent, Belgium; ^2^Department of Ophthalmology, Ghent University Hospital, 9000 Ghent, Belgium; ^3^Department of Genetic Engineering and Biotechnology, Shahjalal University of Science and Technology, Sylhet 3114, Bangladesh

## Abstract

The knowledge on the genetic etiology of complex disorders largely results from the study of rare monogenic disorders. Often these common and rare diseases show phenotypic overlap, though monogenic diseases generally have a more extreme symptomatology. *ABCC6*, the gene responsible for pseudoxanthoma elasticum, an autosomal recessive ectopic mineralization disorder, can be considered a paradigm gene with relevance that reaches far beyond this enigmatic orphan disease. Indeed, common traits such as chronic kidney disease or cardiovascular disorders have been linked to the *ABCC6* gene. While during the last decade the awareness of the wide ramifications of *ABCC6* has increased significantly, the gene itself and the transmembrane transporter it encodes have not unveiled all of the mysteries that surround them. To gain more insights, multiple approaches are being used including next-generation sequencing, computational methods, and various “omics” technologies. Much effort is made to place the vast amount of data that is gathered in an integrated system-biological network; the involvement of *ABCC6* in common disorders provides a good view on the wide implications and potential of such a network. In this review, we summarize the network approaches used to study *ABCC6* and the role of this gene in several complex diseases.

## 1. Introduction

In the western world, the most frequent health problems occur due to complex disorders, such as diabetes, obesity, cancer, and cardiovascular disease [1]. The traditional paradigm for the etiology of these common diseases includes a role for (common) genetic variants, which interact with environmental risk factors, together often coined as a multifactorial etiology [2]. However, dissecting the genes and mechanisms involved in complex diseases has only yielded limited successes, with several reasons for these shortcomings. First, many variants increase the susceptibility to a disease but their individual risk is often too low to be detected by currently used methods. Second, the genetic variants usually require environmental triggers, which need to be taken into account in the search for the underlying genetic causes. As a consequence of these hurdles, the study of rare monogenic disorders, for many of which the genetic causes have been successfully identified in the past, is often said to be a good prerequisite for the understanding of complex disorders since they may show phenotypic overlap with common health problems [3]. However, given the generally more extreme phenotypes of these monogenic disorders, they seem to be easier to study, since they often represent a model of dysfunction of a single biological pathway. Besides pathophysiological insights, knowledge, translated from a rare disease to a more common health problem, can also include innovative targets or options for treatment.

In this review, we exemplify the added value of studying a rare disease, that is, the ectopic mineralization disorder pseudoxanthoma elasticum (PXE, OMIM#264800), and more specifically its causal gene* ABCC6*, for a wide range of more common health as stated in the following list:

(1) Renal disease:
  (i) chronic kidney disease,
  (ii) nephrocalcinosis.
(2) Cardiovascular disease:
  (i) coronary heart disease,
  (ii) cardiomyopathy,
  (iii) dyslipidemia.
(3) Ophthalmological disease:
  (i) age-related macular degeneration.
(4) Cerebral disease:
  - ischemic stroke.
(5) Hematological disease:
  (i) beta-thalassemia.

The use and integration of different research tools (computational, molecular, and biological) in patients, animal, and* in silico* models are emphasized. It is highlighted that proteins which are best known for their association with a rare genetic disorder can have much wider ramifications and that studying them not only benefits patients suffering from rare disorders but also extends the insights in the genetic factors, cell signaling, and (future) therapies in common health problems, hence improving the welfare of a much larger patient population.

## 2. ABCC6

**Figure 1:**
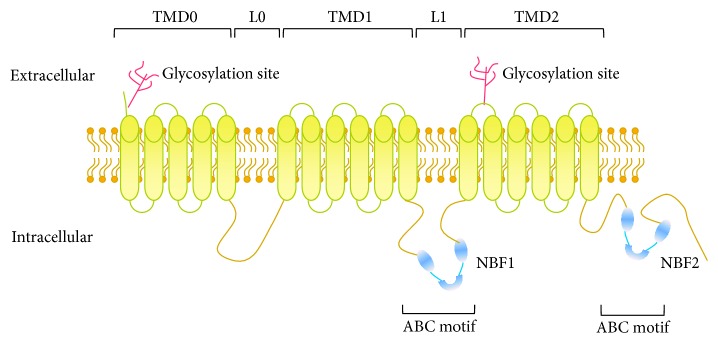
ABCC6 topology model. ABCC6 is a transmembrane transporter, consisting of 3 transmembrane domains (TMD0–2) and 2 linker regions: the latter are built up by the 2 ABC motifs with the 2 nucleotide-binding folds (NBF1-2). Furthermore, there are 2 glycosylation sites in the extracellular part of the transporter.

The ABCC6 (adenosine triphosphate-binding cassette, subfamily C, member 6; OMIM#603234) transporter, also known as MRP6 (Multidrug Resistance Protein 6), is a transmembrane transporter belonging to the superfamily of 49 ABC transporters (Figure 1) [4, 5]. Though it is long known that ABCC6 is primarily expressed in the liver and kidney, to date, the substrate(s) of this transporter remain to be elucidated [6]. Despite marked structural resemblance with other ABC transporters, ABCC6 is considered the odd one out as mutations in the* ABCC6* gene, by which it is encoded, cause PXE, an autosomal recessive connective tissue disease primarily characterized by mineralization and fragmentation of elastic fibers (EFs) in the extracellular matrix (ECM) [4, 7, 8]. Surprisingly, ectopic calcification primarily occurs in soft tissues with minimal expression of the ABCC6 transporter, suggesting that it has a pivotal metabolic role [4, 8]. While ABCC6 is abundantly present in the liver and kidney, PXE patients present a complex phenotype (highly variable in clinical severity) consisting of skin, eye, and cardiovascular symptoms [9, 10]. The skin develops papular lesions, increased skin laxity, and skin folds, mainly in the flexural areas of the body. Ophthalmological manifestations are due to EF fragmentation in Bruch's membrane, a layer which is localized in between the retinal pigment epithelium and the choroidal capillaries, causing a retinopathy characterized by choroidal neovascularization, hemorrhage, and vision loss [11, 12]. The cardiovascular symptoms mainly consist of peripheral and coronary artery disease, which resemble but are not identical to atherosclerosis [13]. As such, the PXE phenotype shows considerable overlap with the symptoms of the common disorders listed in Section 1. More recently,* ABCC6* mutations were also shown to cause generalized arterial calcification of infancy (GACI, OMIM# 208000), featuring extensive vascular calcification and vessel fibrosis in childhood [14].

**Figure 2:**
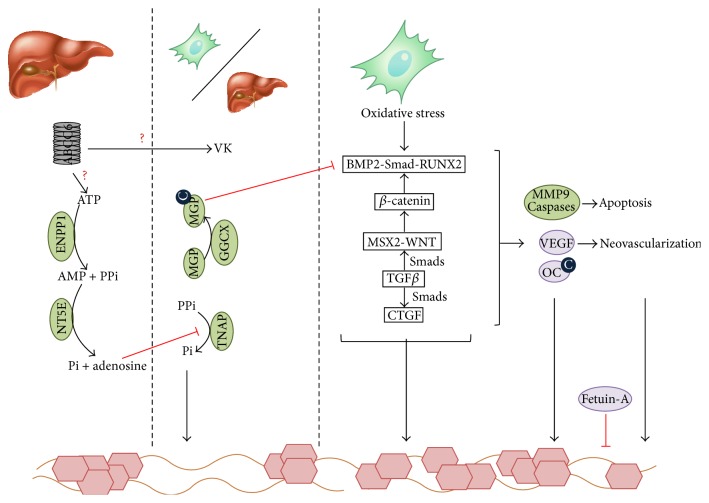
Putative biological pathways related to ABCC6. ABCC6 is mainly expressed in the liver (and the kidneys) and has currently unknown substrates. Furthermore, the molecular mechanisms underlying PXE are still widely unknown but are traditionally explained by the metabolic hypothesis, focusing on the hepatic localization of ABCC6, and the cellular hypothesis, focusing on the role of fibroblasts and peripheral cells. Both are however not necessarily mutually exclusive as shown in this scheme. In the liver, it was shown that ATP is secreted via an ABCC6-dependent mechanism (although ABCC6 itself does not transport ATP). ABCC6 deficiency leads to a hampered transport function, causing a strongly reduced PPi level, which normally inhibits hydroxyapatite growth and hereby mineralization [24]. Further, in PXE, a lower vitamin K (VK) concentration is seen. VK is an essential cofactor of *γ*-carboxylation, which is necessary to activate antimineralizing proteins, such as OC and MGP. Ineffective activation of MGP and OC in peripheral cells also leads to a tissue-specific loss of inhibition of ectopic mineralization together with the diminished levels of the systemic calcification inhibitor fetuin-A [25–27]. Next to these findings, an upregulation of 3 proosteogenic pathways, that is, MSX2-WNT, BMP2-RUNX2, and TGF*β*-CTGF, was found in all PXE-affected tissues compared to controls, which further enhance the ectopic mineralization. These pathways are also associated with altered MMP9 and caspase regulation, leading to apoptosis, and altered VEGF regulation, inducing neovascularization [15]. ABCC6: adenosine triphosphate-binding cassette, subfamily C, member 6; AMP: adenosine monophosphate; ATP: adenosine triphosphate; BMP2: bone morphogenetic protein 2; C: carboxyl; ENPP1: ectonucleotide pyrophosphatase/phosphodiesterase 1; GGCX: *γ*-glutamyl carboxylase; MGP: matrix Gla protein; MMP9: matrix metalloproteinase; MSX2: muscle segment homeobox,* Drosophila*, homolog of, 2; NT5E: ecto-5-prime nucleotidase or CD73; OC: osteocalcin; Pi: inorganic phosphate; PPi: inorganic pyrophosphate; RUNX2: runt-related transcription factor; Smad: mothers against decapentaplegic,* Drosophila*, homolog of; TGF*β*: transforming growth factor *β*; TNAP: tissue-nonspecific alkaline phosphatase; VEGF: vascular endothelial growth factor; WNT: wingless-type MMTV integration site family.

**Figure 3:**
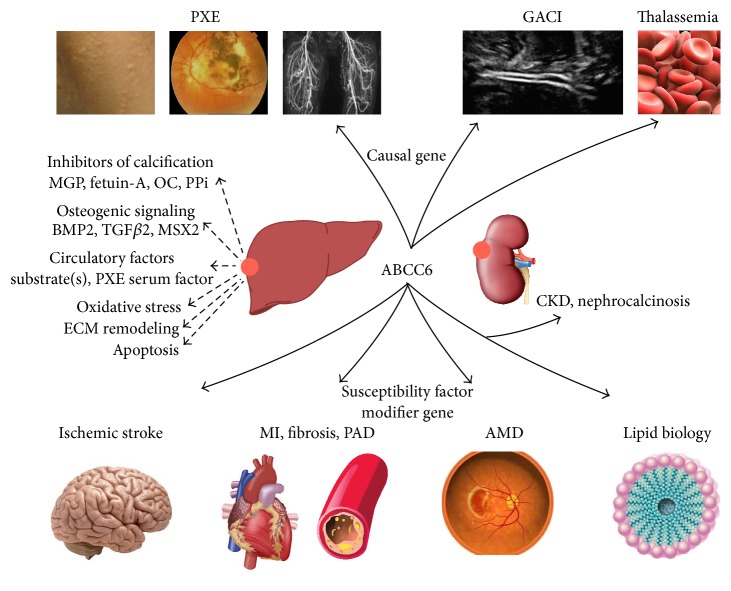
Review of the disorders and pathophysiological mechanisms associated with the ABCC6 transporter, which is primarily expressed in the liver and kidney. Chronic deficiency of the transporter is involved in rare disorders such as pseudoxanthoma elasticum (PXE), generalized arterial calcification of infancy (GACI), and PXE phenocopies in thalassemias. More acute ABCC6 deficiency is a susceptibility factor and/or a modifier for stroke, myocardial infarction (MI), cardiac fibrosis, peripheral artery disease (PAD), age-related macular degeneration (AMD), chronic kidney disease (CKD), nephrocalcinosis, and dyslipidemia. BMP2: bone morphogenetic protein 2; MGP: matrix Gla protein; MSX2: muscle segment homeobox 2; OC: osteocalcin; PPi: inorganic pyrophosphate; TGF: transforming growth factor [4, 7–9, 12, 14, 28–45].

Despite the disadvantage of not knowing the substrates, important progress has been made in the unraveling of the mechanisms in which ABCC6 is involved (Figure 2). To this end, a combination of several approaches is being used, including next-generation sequencing, computational methods, and various “omics” technologies. In doing so, ABCC6 was found to play a role in more pathophysiological processes than could initially be envisaged based on its association with PXE. From this perspective, the ABCC6 transporter is becoming increasingly important for a growing number of diseases (Figure 3).

## 3. A Network Approach towards a Better Understanding of the ABCC6 Transporter

**Figure 4:**
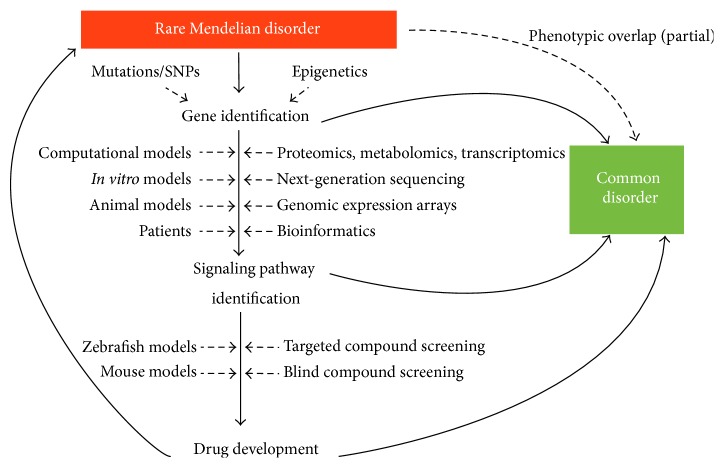
Schematic representation of the multistep process starting from a rare Mendelian disease such as PXE and subsequent identification of the causal gene(s). From then, a combination of technological approaches is performed in several model systems to gain insights into cellular signaling. This knowledge can already be of relevance for common disorders, which show an overlap with the phenotype of the rare disease, but can also be used for development of innovative treatments, which benefit patients of both orphan and complex disorders [20, 21, 46–54].

Over the past years, the field of genetics and genomics has experienced a revolution in the way we think about biological mechanisms and cellular signaling, which for an important part is due to remarkable technological advances. Increasingly, scientific exploration of diseases uses a diverse repertory of approaches to gain insights into the biology underlying a specific disease or protein. These approaches include next-generation sequencing, expression assays, proteomics, transcriptomics, metabolomics, network analysis, and computational biology. Besides the challenges of interpreting the large datasets that are gathered by some of these high-throughput technologies, the integration of all this knowledge into a unified system-biological structure is particularly challenging (Figure 4).

ABCC6 is a prime example on how different approaches, each with its own benefits and particularities, are being used to further unravel this enigmatic protein. However, considering that the substrate(s) of the transporter remain unknown despite more than 15 years of research, the focus has shifted from the protein itself to the potential downstream effects of ABCC6 deficiency [15], which may give valuable clues to its function. In the following paragraphs the most commonly used model systems and techniques in ABCC6 research will be discussed.

### 3.1. Molecular Analysis Tools

The gold standard for molecular analysis of ABCC6 is Sanger sequencing, which can be complemented with a Multiplex Ligand Probe Amplification (MLPA) assay to detect deletions and duplications that would be missed with conventional sequencing [16, 17]. Depending on the cohort, mutation detection rates vary from 70 to 90%, with most mutations located in the 3′ coding region of the gene [16, 17]. A public database enlisting the reported mutations is available at the Leiden Open Variation Database (LVOD, http://www.ncbi.nlm.nih.gov/lovd/home.php?select_db=ABCC6), while neutral variants can be found in dbSNP (http://www.ncbi.nlm.nih.gov/SNP/).

More recently, next-generation sequencing (NGS) has been evaluated as a diagnostic strategy for detecting mutations in* ABCC6* and related genes such as ectonucleotide pyrophosphatase/phosphodiesterase 1 (*ENPP1*, OMIM^*∗*^173335) and gamma-glutamyl carboxylase (*GGCX*, OMIM^*∗*^137167) [18]. Targeted analysis of whole exome sequencing data yielded a mutation detection rate comparable with that of Sanger sequencing with an overall coverage of 37 and over 90% coverage of all* ABCC6* exons. Annotation of variants was done with in-house developed software which uses Ensembl API and Alamut Batch, while filtering of relevant variants was done using* in silico* tools as specified below. To detect false positives, all variants were confirmed with Sanger sequencing. Besides the absence of false positives, it was interesting that also intragenic deletions, considered one of the pitfalls of NGS, could be detected with this approach, possibly making MLPA redundant in the future.

More than 60% of the reported* ABCC6* mutations are missense variants; until now, causality of these variants has been assumed based on their absence in SNP databases and large exome or genome datasets, the conservation of the affected nucleotide and amino acid, the presumed changes in physicochemical properties, and* in silico* prediction tools such as SIFT, PolyPhen, and MutationTaster (Table 1) [16]. It is however well known that the predictions of which variants are deleterious or tolerated made by the latter are often not congruent with reality and should ideally be confirmed with* in vitro* or* in vivo* functional studies. So far, only limited functional data are available proving causality of missense variants. In 2002, Iliás et al. used an* in vitro* modeling system in Sf9 (*Spodoptera frugiperda*) insect cells to establish that 3* ABCC6* missense mutations lead to a loss of transport activity (supplemental Table 1 in Supplementary Material available online at http://dx.doi.org/10.1155/2015/648569) [19]. Since then, consequences of missense mutations have also been tested* in vivo* in a C57BL/6J mouse model, that is, a substrain of the C57BL/6 or “black 6” mouse model, which examined the subcellular localization of the mutant protein in the liver [20, 21]. It has been demonstrated that some missense mutations lead to retainment of the protein in the cytoplasm, whilst others allow the protein to target its physiological location in the plasma membrane (supplemental Table 1). The former could in some cases be rescued using 4-phenylbutyrate which is known to restore intracellular trafficking. More recently, an additional 7 missense mutations were proven to be causal using rescue experiments in an* abcc6a* morpholino-based zebrafish model (supplemental Table 1) [22]. The phenotype of the morphant could not be rescued with coinjected human* ABCC6* mRNA carrying one of these variants, thus demonstrating their deleterious effect.

With the advent of the CRISPR-Cas9 technology as a novel paradigm for genome editing, it can be anticipated that additional functional data of known missense mutations and variants of unknown significance will become available. This ground-breaking technology delivers the Cas9 protein, a nuclease that has the potential to cleave DNA together with specific guide RNAs to the cell and can cut the genome at the desired location [23]. Besides an accurate idea on the actual mutation detection rate, the emerging possibilities of mutation-specific therapies, such as 4-phenylbutyrate, will increase the need for reliable functional data [21].

### 3.2. *In Vivo* Models (Animal Models)

Animal models are used in fundamental research, because of the ethical dilemmas often associated with the use of human tissues for research that does not have immediate therapeutic consequences. Specifically for ABCC6, the paucity of patient tissue due to difficulties with prelevation (e.g., for the liver or eye) also presents an important hurdle. Because of significant gene conservation (depending on the species) between humans and the frequently used mice and more recently zebrafish (ZF) models, animal models can be a valid alternative for these human tissues.

#### 3.2.1. Mice (Mus musculus)

In mice the Abcc6 protein is also predominantly expressed in liver and kidney, though renal Abcc6 is considerably lower compared to humans [28, 55, 56]. Thus far, 2* Abcc6* KO mouse models have been created, one through deletion of the nucleotide-binding fold (NBF1) coding region (Figure 1), a hotspot for* ABCC6* mutations, and a second model via targeted ablation of introns 14–18 [28, 57]. Both models show phenotypic overlap, with spontaneous calcification of EFs (mainly in arteries, the renal cortex, and the capsule surrounding the sinuses of the vibrissae) and normal plasma mineral levels [28, 57]. The prominent vibrissae calcification has been suggested to be a useful biomarker for the phenotype of these mice, though there is no consensus about its relevance for PXE patients [57, 58]. Distinct features of the targeted ablation model include mineralization of dermal EFs and collagen, lower plasma HDL cholesterol, and increased plasma creatinine [28, 57].

A third mouse model that mimics the* Abcc6* ^−*/*−^ phenotype is the dystrophic cardiac calcification (DCC) or* Dyscalc* mouse model [29, 59]. This model harbors an* Abcc6* missense mutation, causing truncation of the protein via the presence of an additional donor splice site. Distinct features are the presence of myocardial calcification and the absence of vascular and vibrissa calcification [29, 60]. Putative explanations for these features are a residual Abcc6 activity in these mice and/or their different genetic backgrounds [29]. Sowa et al. studied the expression of osteogenic factors in the* Dyscalc* mice and found an upregulation of Runx2 in these mice compared to control mice [61]. This is concordant with the findings of Hosen et al. in the* Abcc6* ^−*/*−^ mouse and confirms the added value and complementary character of this murine model [15].

Recently, 4 inbred strains were identified with a spontaneous, nonsynonymous SNP with interference of the* Abcc6* splicing in the liver, which is the primary expression site of murine* Abcc6*. These models, that is, KK/HIJ, 129SI/Svlmj, C_3_H/HeJ, and DBA/2J, show highly variable mineralization phenotypes, rendering them interesting for studying ectopic mineralization (Table 2) [60, 62–64].

Despite the expression profile and the phenotypic resemblance, murine models also present limitations such as their relatively long development time and expensive and labor-intensive maintenance, laborious genome editing and, specifically for PXE, a relatively slow onset of the disease [65]. For these reasons, alternative model systems were strived for.

#### 3.2.2. Zebrafish (Danio rerio)

The ZF was introduced as a novel animal model for ABCC6 research, because of several important advantages compared to mouse models. These include its small size, relative low cost and its fecundity (approximately 100 embryos/week can be generated by 1 ZF pair), its rapid development, and the transparency of the embryos and early adults, which makes it easier to study the internal organs, using light microscopy [65–67]. Because of high conservation of key regulators for skeletal development and calcium metabolism compared to mammals, with significant sequence similarities and overlap in expression between orthologues, ZF are considered a good model system to study mineralization. A potential disadvantage of ZF, specifically for the ABCC6 research, is the presence of 3* abcc6*-related sequences:* abcc6a*,* abcc6b*, and* abcc6c*. This is due to a genome duplication event that occurred during evolution of this species, resulting in a large number of ZF genes to be present in 2 or more copies, which may possibly complicate function analysis [68, 69]. However, in some genes, the ancestral gene function was partitioned between the descendant genes, making it possible to study several aspects of the ancestral (and sole human) gene functions separately [70].

Li et al. characterized a morpholino knockdown ZF model, targeting the* abcc6a* isoform, which developed pericardial edema, a curved tail, and stunted growth, but no ectopic mineralization, possibly due to the early demise of the morphants. Indeed, all embryos died within the first week of life, suggesting that in ZF abcc6a is an essential protein for normal development [67]. Besides this morphant, also a permanent mutant ZF has been studied which shows extensive hypermineralization of the skeleton [71].

The availability of a ZF model for PXE opens novel avenues for functional evaluation of* ABCC6* mutations (see Molecular Analysis Tools) and for the screening of potential therapeutic compounds [66, 72, 73].

Compound screening, that is, a screening to assess the physiological and possible therapeutic effects of chemical compounds by adding them to the water in the ZF tank, can be done using different assays and via a targeted or a blind approach. The latter is done via simultaneous testing of multiple compounds from a compound library. Both approaches are complementary, as a targeted approach often is unsuccessful due to the development of compounds with bad absorption, distribution, metabolization, excretion, and toxicology (ADMET) properties [65]. Due to the advantages inherent to ZF, compound screening in ZF has become easier and faster than it was using other animal models, such as the mouse and even* Drosophila* [65, 72, 73]. Even though the mouse model is still considered more relevant for humans than ZF, it is much more difficult and expensive to manipulate mouse embryos, which need a uterine environment to develop properly and are much more expensive to produce in large numbers compared to ZF embryos [65, 72].

Currently, no blind compound screenings have been performed for Abcc6, but a targeted approach with 4-phenylbutyrate (4-PBA) was successfully applied to rescue the phenotype caused by specific missense mutations. 4-PBA is a chemical chaperone drug that has already been successfully used in cystic fibrosis to restore the plasma membrane localization of CFTR (cystic fibrosis transmembrane conductance regulator, OMIM^*∗*^602421) in the presence of the frequent Δ508 mutation. A major advantage of 4-PBA is that it is already approved by the US Food and Drug Administration (FDA) for other indications (urea cycle disorders and thalassemia) [21, 49–51]. For 7* abcc6* missense mutations which led to mislocalization of the abcc6 transporter but with intact transport activity, 4-PBA could restore the plasma membrane localization in 4, confirming* in vivo* what was previously shown* in vitro* in mouse liver tissues [20]. This suggests that an allele-specific treatment may be interesting in PXE (and GACI). Further, in the permanent mutant PXE ZF model treatment with vitamin K supplementation was evaluated [71]. Interestingly, such treatment was able to rescue the phenotype. This is in contradiction with previous experiments in* Abcc6* ^−*/*−^ mice, in which vitamin K did not have any effect on the mineralization phenotype [74–76]. These results suggest that there are species-dependent peculiarities with regard to ABCC6 and lead to speculating on the reliability of either the ZF or mouse experimental results for the potential treatment of PXE patients with vitamin K supplementation.

### 3.3. *In Vitro* Models

Besides animal models, several* in vitro* cell models are being used to study the physiological roles of the ABCC6 transporter and for evaluating therapeutic interventions. The most reliable cell models remain those derived from patient tissues of which skin fibroblasts remain the most frequently used. For many of the tissues of interest to study the physiological properties of ABCC6, such as the liver or kidney, human cell samples are rare and other* in vitro* model systems are therefore applied. Among these, the most frequently used are Sf9, MDCKII, HepG2, and HEK293 cells.

#### 3.3.1. Patient-Derived Cell Systems

The patient-derived cells, most frequently used to study the function and underlying etiopathogenetic mechanisms of ABCC6, are dermal fibroblasts. In the 70s and 80s multiple studies showed that serum from cultured PXE fibroblasts had a higher protease activity than serum from controls [77, 78]. Later, Le Saux et al. found that human fibroblasts and smooth muscle cells form EF aggregates when maintained in serum from PXE patients, suggesting the presence of disease-specific components in PXE serum [79]. Furthermore, abnormal cell-cell and cell-matrix interactions and a modified proliferative capacity were identified in fibroblasts from PXE patients [79]. This fed the hypothesis of PXE as a metabolic disease, in which the defective ABCC6 transporter is unable to secrete one or more substrates into the serum. More recently, knowledge on mediators involved in ABCC6-related mineralization was derived from dermal PXE fibroblasts, using metabolomics and proteomics technologies (see below) and expression arrays [46, 80, 81].

Furthermore, MMP2 was found to be upregulated in PXE fibroblasts; MGP was found to be highly uncarboxylated and thus inactive to counteract soft tissue mineralization; multiple pro-osteogenic pathways, that is, the BMP2-Smad-RUNX2 (BMP2: bone morphogenetic protein 2, OMIM^*∗*^112261; Smad: mothers against decapentaplegic,* Drosophila*, homolog of, OMIM^*∗*^601366; RUNX2: runt-related transcription factor 2, OMIM^*∗*^60021) and TGF*β*2-Smad2/3 WNT pathways (TGF*β*2: transforming growth factor, *β*2, OMIM^*∗*^190220) and the MSX2-canonical WNT (MSX2: muscle segment homeobox,* Drosophila*, homolog of, 2, OMIM^*∗*^123101; canonical WNT: wingless-type MMTV integration site family, OMIM^*∗*^164820), were found to be upregulated in fibroblasts from PXE patients and were associated with an abnormal cholesterol and lipoprotein metabolism, reinforcing the higher cardiovascular risk found in PXE patients [15, 25, 81, 82]. Very recently, Dabisch-Ruthe et al. identified a strong reduction of the inorganic pyrophosphate (PPi) levels in dermal PXE fibroblasts leading to ectopic mineralization. This could be reduced by PPi addition, confirming what had been shown earlier in primary hepatocytes and* in vivo* liver perfusion experiments [24, 83]. Fibroblasts have also been used to investigate the possible substrates of ABCC6, via immunohistochemistry assays and qPCR experiments and via fluorescence assays in the absence and presence of inhibitors/competitors of ABCC6; however, these experiments have thus far mostly been unsuccessful [84, 85]. Besides the metabolic hypothesis, fibroblast studies have been at the basis of the cellular hypothesis of PXE, with the presence of an increased oxidative stress and a higher susceptibility to procalcifying stimuli [86, 87]. Furthermore, abnormal cell-cell and cell-matrix interactions and a modified proliferative capacity were identified in fibroblasts from PXE patients [88–91].

#### 3.3.2. Non-Patient-Derived (Non)human Cell Lines

Sf9 insect cells are a clonal isolate of* Spodoptera frugiperda* Sf21 cells, which were originally established from ovarian cells [92]. This cell line is commonly used for recombinant protein and after expressing human kidney ABCC6 cDNA on isolated membranes of Sf9 cells using a viral vector (such as a baculovirus vector or pBluescript SK vector) it was shown that human ABCC6 has a high capacity drug-stimulated ATPase activity and that it actively transports leukotriene C(4) and N-ethylmaleimide S-glutathione (NEM-GS) [19, 93]. This transport capacity was inhibited by organic anions (probenecid, benzbromarone, and indomethacin) and 3 PXE-causing missense mutants, although a normal MgATP binding was still possible, indicating that human ABCC6 is a primary active transporter for organic anions [19]. However, the transport rate of these* in vitro* substrates was low and it was deemed that these could not be the physiological compounds transported by ABCC6 [19]. More recently, the observation of low serum vitamin K levels in PXE patients led to the use of this Sf9cell model to show that ABCC6 did not transport the glutathione conjugate of vitamin K3 (VK3GS) [94].

In subsequent studies, experiments in Sf9 cells were often complemented with MDCKII cells (polarized Madin-Darby canine kidney II renal epithelial cells), particularly as in the latter the subcellular localization of ABCC6 at the basolateral cell membrane and N-glycosylation sites were discovered [95]. Further refinement of expression studies, using not only the complete human ABCC6 but also truncated forms (with, e.g., only the TMD0 or the L0), revealed that the TMD0 region is not required for ABCC6 transport function (formation of a transition-state intermediate or nucleotide trapping and leukotriene C4 and N-ethylmaleimide glutathione transport) nor for its proper routing to the plasma membrane [96]. Similarly, it was noted that glycine residues in the ABC motifs and catalytic domain of ABCC6 were of particular importance for its transport function as mutations affecting such conserved glycine residues induced a decrease in ATPase activity, though the ATP binding properties were preserved [97]. Moreover, nucleotide trapping was still possible in these mutants although it was inhibited by ABCC6-substrate drugs, which was possibly due to a miscommunication between the NBFs and the catalytic domains (within the ABC motifs) in ABCC6 [97].

MDCKII cells have also been successfully used to evaluate the therapeutic potential of 4-PBA to overcome incorrect plasma membrane trafficking due to ABCC6 missense mutations [20].

As an alternative kidney cell model, HEK293 cells (Human Embryonic Kidney 293 cells) are used. Jansen et al. showed that medium from these HEK293 cells in which rat or human ABCC6 was overexpressed inhibited mineralization* in vitro* in the chondrogenic cell line ATDC5 (derived from teratocarcinoma AT805), whereas medium from HEK293 control cells did not. This suggests that in serum derived from PXE patients promineralizing factors are present inducing this* in vitro* mineralization, as Le Saux et al. concluded before on fibroblasts [47, 79].

Besides kidney cells, the expression profile of ABCC6 also led to the use of HEPG2 cells as an* in vitro* model system to study polarized human hepatocytes, particularly because of the paucity of patient-derived tissue from this organ. ABCC6 knockdown in HepG2 cells to study mineralization revealed that the promineralizing factor TNAP was upregulated and antimineralizing factors NT5E (CD73), SPP1, and fetuin-A were downregulated [26].

### 3.4. Metabolomics

Metabolomics is the study of metabolites, which are involved in biological processes in cells, tissues, and organisms. Several methodological approaches can be used, such as capillary electrophoresis and High Performance Liquid Chromatography (HPLC). For ABCC6, most metabolic profiling was done using gas chromatography with mass spectrometry. Untargeted metabolic profiling via mass spectrometry comparing human dermal fibroblasts from PXE patients with healthy controls showed substantial alterations in levels of fatty acids, leucine dipeptides, proline oligopeptides, the polypeptide Ac-Ser-Asp-Lys-Pro-OH (AcSDKP), and of pantothenate in cell lysates from PXE patients. These alterations are linked to a.o. cytoskeleton and ECM reorganization, atherogenesis, and angiogenesis, all of which can be localized in the extracellular compartment. Moreover, decreased levels of pantothenate are associated with an increased oxidative stress which, together with ECM remodeling, is a hallmark of the PXE pathogenesis [48]. Jansen et al. also used an untargeted metabolic approach via mass spectrometry, showing an accumulation of nucleoside triphosphates in the supernatant of ABCC6-overexpressing HEK293 cells. Unfortunately, LC-MS experiments on plasma of* Abcc6* ^−*/*−^ and control mice did not confirm the difference between these metabolites. Although ATP is not directly transported by ABCC6, the transport is probably ABCC6-dependent. Furthermore, in this serum an increase in the extracellular PPi levels, a potent antimineralizing agent, was found.* In vitro* and* in vivo* models showed that PPi concentration was significantly lower when ABCC6 was deficient, thus leading to ectopic mineralization [47].

### 3.5. Proteomics

A proteomics approach studies the structure, function, and interactions of proteins produced by genes in a particular organism, tissue, or cell. This method was applied by Boraldi et al. who compared protein profiles in dermal fibroblasts between PXE patients and controls, using 2D gel electrophoresis coupled to mass spectrometry. They identified a decreased protein disulfide isomerase (PDI) level, a protein that is localized in the endoplasmic reticulum (ER) and that is important for proper functioning of the vitamin K cycle. A decrease in PDI leads to a deficient function of vitamin K-dependent processes such as *γ*-carboxylation, which is an essential step for the activation of certain proteins, for example, of matrix Gla protein (matrix *γ*-carboxyglutamic acid or MGP, OMIM^*∗*^154870). Loss of *γ*-carboxylation of MGP, which is specifically linked to EFs, leads to a loss of inhibition of EF mineralization. Furthermore, an increase in calumenin levels, having an inhibitory effect on *γ*-carboxylase, was seen in the ER. Apart from perturbation of the vitamin K cycle, a significant upregulation of superoxide anions (O_2_^−^) production and mitochondrial superoxide dismutase (Mn-SOD) activity as well as an increase in the extracellular SOD (EC-SOD) concentration was found in PXE fibroblasts compared to controls, which can be linked to increased oxidative stress. Moreover, downregulation of the chaperone protein heat shock protein 60 (Hsp60 or HSPD1, OMIM^*∗*^118190), which is required for the correct folding of polypeptides under normal and stress conditions, was identified. This indicates that PXE fibroblasts may be less reactive to tissue injury [46]. The differentially expressed proteins can be targets for validation and efficacy testing of future therapies in PXE fibroblasts (targeted compound screening; see above) [46]. It must be noted that thus far most of the findings from the metabolomics and proteomics studies, such as the involvement of oxidative stress and MGP in the ABCC6-related biological mechanisms, mostly validate data obtained from previous* in vitro* and* in vivo* experiments. This is not surprising, since the data obtained through these untargeted experimental approaches was analyzed mainly in a hypothesis-driven, targeted manner.

### 3.6. Homology Modeling and Molecular Docking

Further insights into the potential substrates of ABCC6 were gained through computational models, such as homology modeling and molecular docking. Homology modeling is the prediction of the structure of a protein based on the structure of a homologous protein (e.g., from the same (super)family). The more the amino acids are conserved between both proteins, the better the prediction of the structure is. Subsequently, molecular docking is an* in silico* technique, used to predict the best orientation of a putative substrate in the binding pocket of a protein in order to form a stable complex [52], in this way helping to find the substrate(s) of a certain protein.

The first ABCC6 homology model was designed by Fülop et al. in 2009, which enabled defining clustering of disease-causing mutations to complex domain-domain interfaces in the ABCC6 transporter. These interfaces include the transmission interface, involving 4 intracellular loops and the 2 ABC domains, and the ABC-ABC interacting surfaces. The mutation prevalence in these regions was 2,75- and 3,53-fold more frequent than the average mutational rates along the ABCC6 transporter. Furthermore, interactions of the mutations within these interfaces were identified [53]. This 3D model was further refined by Hosen et al. and led to better insights into the binding of reported* in vitro* substrates to the ABCC6 protein, by identifying two substrate binding pockets. Blind docking experiments in the open and closed conformation of the protein have enabled generating a list of possible substrates from the Human Metabolome database [54]. These* in silico* modeling techniques can contribute to the discovery of the putative ABCC6 substrate(s), which can then be further tested* in vitro* and* in vivo* [54].

### 3.7. Towards an Integration of Experimental Data

Following the wide variety of approaches used to gain insights into the ABCC6 transporter and the large number of observations related to this transporter, to date, one of the main challenges remains to integrate all these observations into a (patho)physiological model of ABCC6. An interesting initiative in this respect is the Clinical and Functional Translation of CFTR (CFTR2) project, which deals with a different ABC transporter, CFTR or ABCC7, causing cystic fibrosis (Castellani and CFTR2 team, 2013). The CFTR2 project aims to integrate clinical, molecular, and functional data on the* CFTR* gene and its encoded protein in a single database, curated by a data manager. Such an initiative stands as an example for a novel cloud-based data management system which is being implemented in Ghent, Belgium, as a prerequisite for a European and international initiative to have a complete overview of research observations on ABCC6 and thus move forward efficiently in understanding this protein.

## 4. Relevance of ABCC6 for Complex and Rare Disorders and Disease Risk Factors

### 4.1. Kidney Disease

#### 4.1.1. Chronic Kidney Disease

Though PXE patients themselves only rarely present nephrological problems, except for the occasional observation of ectopic calcification foci in the kidney parenchyma, the vascular tunica media calcifications in PXE show a striking resemblance with vascular disease seen in chronic kidney disease (CKD) patients: fragmentation of EFs is present in the aortic wall of CKD mice and fragmentation of medial EFs has been described in CKD patients [98–100]. This arterial disease contributes more to the high prevalence of cardiovascular morbidity and mortality than does the development of end-stage kidney failure [101]. In the dialysis population, mortality risk is most marked in the younger age group (25–34 years old), where the cardiovascular death rate is up to 500-fold greater than in the age-matched control group [30]. Several mediators have been discovered which are involved in CKD-related vascular disease, promoting soft tissue mineralization and accelerating arterial calcification. These include systemic inflammation, altered calcium and phosphate homeostasis, hypertension, and a deficiency of endogenous calcification inhibitors such as Klotho, MGP, PPi, and fetuin-A [30, 102]. However, common mechanisms underlying these pathophysiological aberrations have not been determined.

Because of the histological resemblance, Lau et al. studied the potential involvement of the Abcc6 transporter in CKD rats and mice and found a significantly decreased level of Abcc6 protein in their liver and kidney tissues [30]. As the Abcc6 mRNA levels were normal, this was likely due to an as yet unidentified posttranscriptional or posttranslational mechanism. However, if extrapolated to the human situation, it suggests that an acquired ABCC6 deficiency contributes to accelerated arterial calcification in CKD patients which may explain the highly prevalent medial calcification in CKD, even in the absence of traditional atherosclerotic risk factors, for example, in pediatric dialysis patients. Indeed, it has been demonstrated in mice with an* ApoE* ^−*/*−^ background that an* Abcc6* deletion results in medial calcification within the vessel wall without calcification or enlargement of existing atherosclerotic lesions [31]. These findings are of interest because in PXE a disturbance of MGP, PPi, and fetuin-A has also been shown and related to the deficient ABCC6 transporter [24, 27]. The involvement of ABCC6 thus gives an explanation for at least some of the pathophysiological observations previously done in CKD. Furthermore, it confirms that the function of the ABCC6 transporter is not restricted to a multisystem calcification disease such as PXE but also contributes to vascular disease without causing any of the other phenotypic features for which it is renowned. Finally, these findings may also have therapeutic consequences. Indeed, overexpression of fetuin-A in* Abcc6* ^−*/*−^ mice has been shown to attenuate soft tissue mineralization [63]. Moreover, recently, the use of bisphosphonates, that is, nonhydrolyzable PPi analogs, has been suggested as a potential treatment for ABCC6-related mineralization, to counteract PPi deficiency [47].

#### 4.1.2. Nephrocalcinosis

A second renal phenotype in which ABCC6 was shown to play a role is nephrocalcinosis. It is characterized by aberrant deposition of calcium in the kidney parenchyma and tubules and can be associated with a number of systemic and renal metabolic diseases, including acute phosphate nephropathy, primary hyperparathyroidism, and distal renal tubular acidosis [103]. The aberrant calcium deposition may be asymptomatic but can eventually lead to progressive renal failure and end-stage renal disease. It has been suggested that nephrocalcinosis could be considered a passive phenomenon resulting from deposition of a supersaturated phosphate product associated with tissue remodeling and ultimately leading to the loss of functional renal parenchyma [104]. However, the mechanisms underlying many of the pathological conditions resulting in ectopic calcium deposition in the kidney remain to be explored.

An association of PXE with nephrocalcinosis was described in a few cases [32]. Though these may have been fortuitous, Li et al. further examined the presence of nephrocalcinosis in two animal models of ectopic mineralization, respectively, the* Abcc* ^−*/*−^ (via targeted ablation) and* Enpp1* ^*asj*^ mutant mouse, an animal model for generalized arterial calcification of infancy, both characterized by multisystem deposition of mineral crystals [57, 105, 106]. These mice develop nephrocalcinosis only at a later age when fed with a normal diet. Treated with a calcification-accelerating diet (containing decreased amounts of magnesium and increased phosphate), these animals develop extensive mineralization in the kidney interstitium, primarily affecting the medullary tubules as well as the arcuate and renal arteries. Interestingly, the heterozygous mutant mice did not develop nephrocalcinosis, while in the compound heterozygous mice mineral deposits in the kidneys were shown to consist of calcium and phosphate, suggesting the presence of hydroxyapatite crystals, which is similar to human nephrocalcinosis [106]. This is the first evidence of a synergistic effect of two defects in mineralization homeostasis modulating soft tissue calcification in a common disease such as nephrocalcinosis.

### 4.2. Cardiovascular Disease

#### 4.2.1. Coronary Heart Disease

Coronary heart disease (CHD) is a major cause of death and disability in our society, causing approximately 450,000 deaths/year in the United States only. Besides environmental factors, there is evidence for a strong genetic predisposition with currently over 50 genetic risk factors identified [107]. Most of these are related to dyslipidemia or have an as yet to be elucidated mechanism. Though in PXE patients CHD is less frequent compared to peripheral artery disease, studies have been conducted to evaluate whether ABCC6 variants may be susceptibility alleles for CHD beyond PXE [9]. Some studies have shown that the frequent R1141X mutation is risk factor for CHD among Caucasians, even in heterozygous state [9, 33, 108]. However, others were not able to show this association, which is in analogy with the absence of a genotype-phenotype correlation between ABCC6 mutations and the PXE phenotype [109]. Thus far no other studies have been done looking at a possible association between the whole* ABCC6* mutation spectrum and CHD instead of focusing merely on a single mutation. A need for such a more global evaluation seems highly relevant as several observations in* Abcc6*-deficient mice also point towards a causal relationship with CHD.

Mungrue et al. found an increased infarct size and apoptosis in a mouse cardiac ischemia-reperfusion model based on a naturally occurring Abcc6 deficiency, suggesting a role for Abcc6 in cardioprotection as a novel modulator of cardiac myocyte survival after ischemia-reperfusion [110]. It was shown that the BMP-responsive transcription factors pSmad1/5/8 were upregulated in the cardiac tissue of these* Abcc6* ^−*/*−^ mice together with an increased propensity to apoptosis. Both are very similar to what is found in PXE patients and suggest that BMP signaling and apoptosis are end results of ABCC6 deficiency [15].

Moreover, the level of hepatic* Abcc6* expression also determined the severity of postinjury calcification, thus making this an important factor in determining myocardial dystrophic calcification [111]. Although less frequent than vascular calcification, myocardial dystrophic calcification occurs in long-term survivors of myocardial infarction with an occasionally extremely severe presentation [112]. These observations should not come as a surprise, given that a splice site* Abcc6* allele was previously found to be the cause of a cardiac phenotype in mice coined dystrophic cardiac calcification or DCC in which myocardial calcification is a hallmark [29]. The results of the experiments support the notion that the substrate(s) of ABCC6 may have a wider therapeutic value, stretching to myocardial infarction. These results also define a novel mechanism of adverse outcome after cardiac injury and again support the usefulness of a broader examination of* ABCC6* variants (both mutations and SNPs) in cardiovascular disease. Similarly, Rau et al. applied genome-wide association studies (GWAS) in mice after *β*-adrenergic stimulation and detected 7 significant loci affecting cardiac hypertrophy, fibrosis, and heart failure, one of which harbored the* Abcc6* gene. Functional evaluation demonstrated that a splice site mutation in* ABCC6* strongly promoted stress-induced cardiac fibrosis, thus corroborating ABCC6 as an unrecognized and novel player in the development of cardiac fibrosis and acquired cardiomyopathy [34].

#### 4.2.2. Dyslipidemia

One of the most important cardiovascular risk factors is dyslipidemia. Population studies have shown that a strong inverse relationship exists between plasma high-density lipoprotein cholesterol (HDL-C) concentrations and CHD risk [113–116]. Indeed, a low level of HDL-C is the most common plasma lipid abnormality observed in men with established CHD [117]. In addition to environmental factors, strong evidence exists for the role of genetics in the determination of HDL-C levels with heritability estimates in the range of 40–60% [118].

Since several ABC transporters are involved in lipid homeostasis, a role for ABCC6 in the lipoprotein metabolism was not surprising and this was corroborated by several studies. Allelic variants in the* ABCC6* gene (rs150468 and rs212077) were found to be associated with susceptibility to low HDL-C and CHD [119]. A haplotype analysis of these two SNPs demonstrated that the haplotype with the AC alleles (frequency of 81% in studied population) had a 56% increased risk to develop CHD/low HDL-C compared to the CG haplotype (frequency of 16%) [119].

Cross-sectional studies involving relatively large numbers of PXE patients indicated a higher than expected prevalence of hypertriglyceridemia and depressed plasma HDL cholesterol [120]. PXE patients with moderately severe type IV hyperlipoproteinemia were screened and it was shown that* ABCC6* p.(R1164X) nonsense mutation and the* ABCC6* p.(R1268Q) SNP are associated with dyslipidemia, characterized by high plasma triglyceride and low HDL cholesterol [35, 36].

Very similar findings were seen in experiments in* Abcc6* ^−*/*−^ mice showing a 25% reduction in plasma HDL-C, confirming the potential role of ABCC6 in lipid homeostasis [28]. The possible relevance of these lipid abnormalities for soft tissue mineralization was demonstrated by Guo et al., who found that atorvastatin counteracts soft tissue mineralization in Abcc6-deficient mice [37]. Very recently, Kuzaj et al. documented increased ABCC6 transcription levels as well as an increased cholesterol biosynthesis in lipoprotein-deficient serum. Furthermore, the serum also contained elevated PCSK9 (proprotein convertase, subtilisin/kexin-type 9, OMIM^*∗*^607786) levels, which cause a decrease of (extra)hepatic levels of LDL-C (low-density lipoprotein cholesterol) receptors and an increase in LDL plasma concentration. In PXE fibroblasts a reduction of ApoE mRNA expression was identified [81]. These findings further stress the importance of* ABCC6* variants, both gain- and loss-of-function, in the cardiovascular disease risk of the general population.

#### 4.2.3. Vascular Aneurysms

An abdominal aortic aneurysm (AAA) is a complex multifactorial disease with a prevalence of 1–6% in industrialized countries, the etiology of which is incompletely understood. This disease is characterized by arterial wall dilatation accompanied by the degradation of elastin and collagen fibers. The most severe complication of this disorder is a rupture of the weakened vessel wall leading to significant mortality and morbidity. While in some individuals there is a clear genetic predisposition, other cases of AAA seem to arise spontaneously [121].

Contrary to other connective tissue diseases such as Marfan syndrome or the vascular type of the Ehlers-Danlos syndrome, in PXE patients aneurysms are sparse if not anecdotic [122]. Aortocoronary aneurysms have also been reported but seem not to be specific to PXE since they have also been reported in other PXE-like phenotypes such as in the PXE phenocopy associated with beta-thalassemia [38, 123].

To date, only 1 study has been conducted, which investigates the potential association of* ABCC6* mutations and AAA. Mutations were found in only a small minority of non-PXE patients (5/133) with AAAs [124]; however, this was not statistically different from healthy controls. Further research would be appropriate before drawing a definite conclusion not to consider* ABCC6* as a genetic risk factor for AAA.

### 4.3. Ophthalmological Disease

#### 4.3.1. Age-Related Macular Degeneration

In the European and North American population age-related macular degeneration (AMD) is a main cause of irreversible, central blindness in patients over 50 years of age. It is a common multifactorial disorder, causing a progressive deterioration of macular function, which is responsible for central vision [125–127]. In an advanced stage, the disease is classified into 2 forms: a dry, nonexudative form and a wet or exudative form. The wet form, being responsible for 90% of the AMD-related vision loss, is characterized by subretinal neovascularization and hemorrhage, which are also hallmarks of PXE [11, 12, 126]. As a prototype multifactorial disease, the pathophysiologic factors in AMD include not only changes in Bruch's membrane but also oxidative stress [128]. In multifactorial diseases, it is difficult to separate primary effects from secondary effects that eventually lead to functional loss. For instance, choroidal alterations could occur, secondary to changes in Bruch's membrane. To investigate such specific components in AMD pathology a detailed investigation of human monogenic disease with clearly defined changes mimicking specific components is very useful.

PXE is a good candidate for studying the impact of altered Bruch's membrane as the primary pathologic alteration in eyes of patients with PXE appears to be a thickening and calcification of this membrane [12, 129]. In later disease stages, the disease shares various phenotypic similarities with AMD, including a high risk for developing choroidal neovascularization or chorioretinal atrophy of the macular region [12]. A significant reduction of mean subfoveal choroidal thickness was found in the eyes of PXE patients. PXE eyes without CNV or chorioretinal atrophy showed the least reduction of choroidal thickness, while it was most pronounced in PXE eyes with chorioretinal atrophy. The results indicate that changes of Bruch's membrane can be associated with choroidal alterations, which are most pronounced in the presence of advanced disease. A role for Bruch's membrane in choroidal homeostasis may reflect a possible contribution of Bruch's membrane alterations to CNV and geographic atrophy development in age-related macular degeneration [12, 130].

### 4.4. Cerebrovascular Disease

#### 4.4.1. Ischemic Stroke

Stroke is a multifactorial and polygenic disease, which has a monogenic basis in only a small percentage of cases. In the general population, the incidence of ischemic stroke under 65 years is 229/100,000/year [131–133]. There is increasing evidence of an important genetic component in the etiology of stroke [134]. Especially in cryptogenic stroke, where no clear cerebrovascular risk factors or triggers can be found to explain the acute event, the identification of one or more genetic variants (either as a monogenic cause or as a susceptibility factor) can have important implications for therapeutic management and genetic counseling.

A large number of single-gene disorders, including PXE, have been described to be associated with stroke. In PXE, ischemic stroke is caused by large artery disease or less frequently by small-vessel occlusive disease [9]. Moreover, hypertension, which is very common in patients with PXE, favors cerebrovascular episodes [135, 136].

The role of heterozygous* ABCC6* variants in cryptogenic ischemic stroke has been investigated in a pilot study in our group. The frequency of* ABCC6* mutations—not limited to the frequent p.(R1141X) mutation—was found to be much more frequent in the stroke cohort compared to controls, with an odds ratio of 5. This would imply that heterozygous* ABCC6* mutations are an important risk factor for the development of ischemic stroke, which would have important implications for the counseling and management of PXE and cryptogenic stroke families. However, Köblös et al. found no association between carriers of the p.(R1141X) mutation and stroke, indicating that further research is needed before a conclusion can be made [33]. Similar to CHD, it is worthwhile to evaluate the complete* ABCC6* mutation spectrum to clarify these conflicting results.

A possible role of ABCC6 in the central nervous system was recently enforced by Wakabayashi et al. who, using flow cytometry, showed the presence of a side population (SP) of endothelial cells in brain vessels, capable of exteriorizing the Hoechst 33342 dye in C57BL/6 and C57BL/6-Tg, a variant of the former strain containing enhanced GFP cDNA that makes all the tissues (except hair and erythrocytes) appear green under excitation light, mouse models. Similar SP cells have already been shown to cause resistance in cancer cells by enhancing the efflux of chemotherapeutics. This feature is linked to a high ABC transporter activity, among which the ABCC6 transporter seems to be of importance, as was shown by its high mRNA levels in the murine brain tissues. Due to their efflux capacity, these SP cells seem to play an important role in brain homeostasis, by contributing to the brain's barrier functions (i.e., the blood-brain barrier) and prevention of cytotoxicity to the brain parenchyma [137]. This suggests that an impairment of the ABCC6 transporter may cause dysfunction of the blood-brain barrier and puts the focus on the role of ABCC6 in the cerebral vasculature.

#### 4.4.2. Spontaneous Carotid Artery Dissections (sCAD)

In a similar way, the frequency of* ABCC6* variants has been studied in patients with spontaneous carotid artery dissections (sCAD). sCAD represents an important cause of stroke among young and middle aged patients and can occur without relevant trauma in otherwise healthy individuals without known risk factors [138]. Although the clinical and diagnostic criteria for sCAD are well established, the pathogenesis is still a matter of speculation. In some patients with sCAD the reticular dermis has a similar, although less severe, morphology to heterozygous relatives of PXE patients with fragmented, cribriform, and mineralized collagen bundles and EFs [139]. However, Morcher et al. were not able to find an association between* ABCC6* variants and sCAD patients with pronounced electron microscopic alterations in the dermal connective tissues [140]. Though it was concluded that* ABCC6* is not a candidate gene for sCAD, we should be careful to draw definite conclusions based on a single study.

### 4.5. Familial Mediterranean Fever

Familial Mediterranean fever (FMF) is an autosomal recessive disorder caused by mutations in the* MEFV* (familial Mediterranean fever gene, OMIM^*∗*^608107) gene and is characterized by recurrent inflammatory attacks, mainly affecting ethnic groups originating from countries around the Mediterranean Sea. FMF shows a marked variability in clinical expression between and within families [141, 142]. Amyloidosis, one of the severe complications of FMF, was relatively frequent before the advent of colchicine as a therapy. The risk of amyloidosis depends on contributing factors, such as ethnicity, nature of the* MEFV* mutation, the* SAA1* (serum amyloid A1, OMIM^*∗*^104750) gene haplotype, and environmental factors [143, 144]. However, these factors do not account for the total contribution to amyloidosis susceptibility. A patient suffering from both FMF and PXE was found to be homozygous for both the p.(Met694Ile) mutation in* MEFV* and p.(Gly1042Ser) in* ABCC6* [145, 146]. As this patient developed severe amyloidosis despite appropriate colchicine treatment, the possibility that ABCC6 deficiency contributed to the severity of FMF was raised. Two nonexclusive pathogenic explanations have been proposed: increased amyloid deposition in target tissues and/or decrease in colchicine activity [145]. Though thus far no validation studies have been performed, the identification of* ABCC6* variants would be important in improving the accuracy of amyloidosis risk prediction in patients and in extending our understanding of the pathophysiology of FMF.

### 4.6. Beta-Thalassemia

Beta-thalassemia is a mostly autosomal recessive disorder caused by mutations in the beta-globin gene, which leads to diminution of the protein concentration. The disease has a highly variable phenotype and is relatively frequent in the Mediterranean population, the Middle East, India, and Southeast Asia. Although beta-thalassemia is not caused by* ABCC6* mutations [39–41], patients may develop an ectopic mineralization phenotype, identical to PXE, which is thus considered a PXE phenocopy [38, 42–44]. Although in humans no apparent link with ABCC6 (dys)function has been demonstrated, there is some evidence in murine models that Abcc6 is involved in the occurrence of this phenocopy. Indeed, perturbation of Abcc6 function was found in a *β*-thalassemia mouse model (*Hbb* ^*th*3/+^), showing a progressive liver-specific downregulation of* Abcc6* gene expression. This downregulation is due to the absence of NF-E2, a transcription factor from the Abcc6 promotor, which is also an important transcription factor involved in the regulation of expression of several hemoglobin-related genes [41, 45]. It should however be noted that these mice did not develop spontaneous calcification, possibly due to the genetic background of these mice (DCC-resistant C57BL/6J) or due to the fact that the Abcc6 decrease only occurred late in life [40, 41].

## 5. Conclusions

The example of ABCC6 shows that genes that are initially discovered as the cause of orphan diseases can have widespread effects with implications for a wide range of common, often complex, diseases. This expands the knowledge on the genetic etiology of these diseases and can have beneficial effects on the diagnosis, follow-up, and management as well as treatment of patients suffering from these diseases. At the same time, ABCC6 exemplifies the complexity of the biological systems that underlie these diseases and the pleiotropy of approaches, which is necessary to unravel the associated (patho)physiology. ABCC6-related research demonstrates the feasibility of translating findings in animal studies to humans; at the same time, it emphasizes the continuous need for thorough and comprehensive studies, for example, studies which investigate not only a single* ABCC6* mutation but the whole mutation spectrum to identify risk alleles for CHD and stroke, certainly as many indications exist for the relevance of ABCC6 in these disorders, as well as replication studies. The main challenge for the future is the so-called multilayered data integration. Specifically for* ABCC6*, we have started with the development of a cloud-based system which is able to document and integrate the clinical, molecular, histological, and biochemical data of individual patients together with their data obtained from proteomics, metabolomics, and expression arrays. As this integrative system (under the control of a data manager) will be open to other research groups, it will be a valuable asset to move forward in understanding the role of ABCC6 in human disease.

## Supplementary Material

Supplemental Table 1. Missense variants in the ABCC6 gene for which causality has been studied by in vitro and in vivo functional studies [19] [20] [21] [22]. SCL: subcellular localization, TA: transport activity, +: normal; -: abolished; ZF: zebrafish mRNA rescue experiment; NA: not available.

## Figures and Tables

**Table 1:** *In silico* prediction tools used to study causality of ABCC6 missense variants.

Bioinformatics tool	Web address
Annotation of variants	
Ensembl API	http://rest.ensembl.org/
Alamut Batch	http://www.interactive-biosoftware.com/alamut-batch/

Functional consequences of mutations	
Alamut	http://www.interactive-biosoftware.com/software/alamut/overview
Polyphen 2	http://genetics.bwh.harvard.edu/pph2/
SIFTs	https://www.ebi.ac.uk/pdbe/docs/sifts/
Align-GVGD	http://agvgd.iarc.fr/agvgd_input.php
MutationTaster	http://www.mutationtaster.org/
SpliceCenter	http://projects.insilico.us/SpliceCenter/SpliceOverview.jsp
MutationAssessor	http://mutationassessor.org/v1

Structural consequences of mutations	
PredictProtein	https://www.predictprotein.org/
MutDB	http://www.mutdb.org

Large sequencing datasets	
1000 genomes browser	http://www.1000genomes.org/
Exome variant server	http://evs.gs.washington.edu/EVS/

**Table 2:** Mineralization phenotypes of PXE-related murine strains (based on [60]).

Strain	Phenotype
129S1/SvImJ	Fibroosseous bone lesions^*∗*^ Vibrissae mineralization

C3H/HeJ	Epicardial fibrosis and mineralization Fibroosseous bone lesions^*∗*^

DBA/2J	Epicardial fibrosis and mineralization Fibroosseous bone lesions^*∗*^ Arterial mineralization

KK/HIJ	Systemic mineralization (lung, retina) Epicardial fibrosis and mineralization Fibroosseous bone lesions^*∗*^ Arterial mineralization Vibrissae mineralization Hyperplasia (most common in pancreatic islets) [24009271]

Abcc6^−/−^ (targeted ablation)	Spontaneous calcification of EFs (mainly in arteries, the renal cortex, and the capsule surrounding the sinuses of the vibrissae) Normal plasma mineral levels Mineralization of dermal EFs and collagen Lower plasma HDL cholesterol Increased plasma creatinine

Abcc6^−/−^ (deletion of NBF1)	Spontaneous calcification of EFs (mainly in arteries, the renal cortex, and the capsule surrounding the sinuses of the vibrissae) Normal plasma mineral levels

DCC or Dyscalc	Myocardial calcification Absence of vascular and vibrissa calcification

^**∗**^This lesion was also found in strains without PXE-like mineralization and was not linked to EF calcification. Probably this lesion is thus not associated with PXE or Abcc6 was a strong modifier gene in KK/HIJ mice when mutated [60]. The other described lesions, apart from hyperplasia in the KK/HIJ mouse, can be linked to PXE. NBF1: nucleotide-binding fold 1.
