# Supplementary material for: The ABCC6 Transporter as a Paradigm for Networking from an Orphan Disease to Complex Disorders
Source: Biomed Res Int. 2015 Aug 18;2015:648569. doi: 10.1155/2015/648569 (PMC4555454; doi:10.1155/2015/648569)
Supplement: Supplementary file 1 — Supplemental Table 1. Missense variants in the ABCC6 gene for which causality has been studied by in vitro and in vivo functional studies [19] [20] [21] [22]. SCL: subcellular localization, TA: transport activity, +: normal; -: abolished; ZF: zebrafish mRNA rescue experiment; NA: not available. [file 648569.f1.pdf]

	Missense variant	Gen Bank Accession No.	Model system	TA	SCL	+
**Ilias et al.**	p.V1298F	AF076622	Sf9 insect cells	-	NA	
	p.G1302R			-	NA	
	p.G1321S			-	NA	
**Le Saux et al.**	p.R1138Q	NM_001171	Sf9 insect cells<br>Retroviral gene delivery in MDCKII cells	+	-	
	p.V1298F			-	+	
	p.R1314W			+	-	
	p.G1321S			-	-	
	p.R1339C			NA	-	
**Pomozi et al.**	p.R1114P	NM_001171	Sf9 insect cells<br>Retroviral gene delivery in MDCKII cells	+	+	-
	p.S1121W			+	-	-
	p.R1138Q			+	-	-
	p.V1298F			-	+	-
	p.T1301I			+	-	-
	p.R1314W			+	-	-
	p.G1321S			-	-	-
	p.R1339C			NA	-	-
	p.Q1347H			+	-	-

	p.R1459C			+	+	-
**Uitto et al.**	p.P4H	NM_001171		NA	+	-
	p.A9E			NA	+	-
	p.P21S			NA	+	-
	p.R64Q			NA	+	+
	p.E125K			NA	+	-
	p.R419Q			NA	+	-
	p.L605P			NA	-	NA
	p.E709G			NA	+	-
	p.M834T			NA	+	NA
	p.L948P			NA	+	-
	p.R1114P			NA	-	NA

Supplemental table 1. Missense variants in ABCC6 for which causality has been studied by *in vitro* and *in vivo* functional studies [19] [20] [21] [22]. SCL: subcellular localization, TA: transport activity, +: normal; -: abolished; ZF: zebrafish mRNA rescue experiment; NA: not available.
